# Case Report: Unmasking sustainable left ventricular recovery in chronic heart failure with axillary temporary mechanical circulatory support

**DOI:** 10.3389/fcvm.2024.1407552

**Published:** 2024-08-27

**Authors:** Aarti Desai, Shriya Sharma, Caitlyn Luce, Jose Ruiz, Rohan Goswami

**Affiliations:** Division of Advanced Heart Failure and Transplant Cardiology, Mayo Clinic, Jacksonville, FL, United States

**Keywords:** Impella, ischemic cardiomyopathy, heart failure, cardiogenic shock, transplantation, mechanical circulatory support, HF-CS

## Abstract

**Background:**

Mechanical circulatory support (MCS), temporary or durable, is essential in patients with acute heart failure presenting in cardiogenic shock (CS). MCS is fundamental in patients with advanced heart failure when used as a bridge to decision, transplant or left ventricular recovery. Limited data on acute-on-chronic heart failure (HF) patients exists in the era of axillary mechanical circulatory support with the Impella 5.5. We describe a case of chronic ischemic cardiomyopathy, HF-CS, in a patient who underwent Impella placement, medical optimization, and explant, now with sustained normalization in ejection fraction.

**Case summary:**

A Caucasian female in her 50 s was referred to our center for evaluation for advanced therapies, including transplantation or durable left ventricular assist device placement. Her initial ejection fraction was 30% with comorbidities including multivessel coronary artery disease revascularized with 3 vessel bypass grafting ten years prior, type 2 diabetes (A1c 8.6%), and peripheral vascular disease. During her evaluation, she had acute decompensation leading to cardiogenic shock and required hospitalization with inotrope initiation, which was unable to be weaned. She was approved for organ transplant and listed; however, she required escalation of support and eventual placement of right axillary Impella 5.5. While on Impella support, her vasoactive needs reduced, and she was found to have left ventricular recovery and tolerated the initiation of guideline medical therapy. After three weeks of support, the Impella was weaned and explanted, and the patient was discharged. She remains stable with a sustained ejection fraction of greater than 50% with NYHA class 1 functional status at follow-up. One year later, the patient showed sustained myocardial recovery with guideline-directed medical therapy (GDMT).

**Conclusion:**

Our case highlights a unique approach in patients with long-standing (>5 years) heart failure who may benefit from early consideration for axillary support and concomitant optimization with guideline-directed medical therapy to assess for explant and native heart recovery.

## 1 Introduction

Cardiogenic shock (CS) is characterized by insufficient cardiac output from myocardial dysfunction, leading to inadequate perfusion of vital organs (1). Individuals experiencing CS are in a critical condition and are susceptible to rapid deterioration.

The role of mechanical circulatory support (MCS) in patients with acute-on-chronic decompensated heart failure is pivotal. It aims to reduce cardiac workload, lower left ventricular (LV) pressures, improve myocardial oxygen consumption, enhance coronary and end-organ perfusion, and reduce pulmonary congestion (2–4). The enhanced cardiac output provides a window of stability, increasing time for organ recovery and determining if medical management, candidacy for advanced therapies, or potential for left ventricular recovery are feasible (2). The currently approved percutaneous mechanical circulatory support (MCS) devices for LV unloading include Intra-aortic balloon pump (IABP), Impella (Abiomed, Danvers, MA), veno-arterial extracorporeal membrane oxygenation (VA-ECMO), and TandemHeart (5).

We highlight the utilization of early MCS with Impella 5.5 leading to recovery in a patient with acute-on-chronic decompensated heart failure (HF) progressing to cardiogenic shock (HF-CS)—an outcome deemed improbable by many due to her chronic ischemic cardiomyopathy. The Impella 5.5 with SmartAssist, a percutaneous microaxial left ventricular assist device (LVAD) implanted via minimally invasive surgery. The device facilitated left ventricular recovery. This support ultimately resulted in reversal of end-organ damage and recovery of native LV function. After Impella explant the patient tolerated guideline-directed medical therapy (GDMT) and was discharged in stable condition.

## 2 Case presentation

A female in her mid-50s with a history of significant multivessel coronary artery disease treated with 3 vessel coronary artery bypass graft ten years prior, HF with reduced ejection fraction at 34%, dual chamber implantable cardioverter-defibrillator, as well as type 2 diabetes mellitus (A1c 8.6%), stage 3 chronic kidney disease (eGFR 32 ml/min/m^2^), hyperlipidemia and body mass index (BMI) 35.8 kg/cm^2^, was referred to our facility for evaluation of advanced heart failure therapies.

She had been experiencing progressively worsening fatigue, shortness of breath, lower extremity edema, and chest pain, leading to significant functional impairment despite receiving maximally tolerated guideline-directed medical therapy on an outpatient basis. This included atorvastatin 80 mg BID, bumetanide 2 mg BID, clopidogrel 75 mg OD, dapagliflozin 10 mg OD, lisinopril 40 mg OD, metoprolol succinate 50 mg OD, entresto 24–26 mg BD, and spironolactone 25 mg OD.

Due to progressive HF symptoms, she was admitted. Repeat 2d transthoracic echocardiogram showed an LVEF of 30% and NT-pro-BNP of 2,253 pg/ml (Table 1). Despite aggressive diuresis with bumetanide and attempts at optimizing inotropes, her hemodynamics failed to improve. A right heart catheterization (RHC) while on Dobutamine 2.5 mcg/kg/min was performed to guide therapy and showed a mean arterial pressure (MAP) of 61 mmHg, right atrial pressure (RA) 17 mmHg, pulmonary artery pressure (PA) 42/31 (35) mmHg, pulmonary capillary wedge pressure (PCWP) of 31 mmHg, Fick cardiac output (CO) of 4.2 L/min and Fick cardiac index (CI) of 1.96 L/min/m^2^. The patient failed to improve on higher doses of inotrope therapy and had worsening kidney function with more aggressive diuresis. Given that the patient remained in cardiogenic shock (CI < 2.2 L/min/m^2^) we felt that utilizing axillary support with Impella 5.5, based on our institutional standard of care, would improve left ventricular unloading, and serve as a bridge to decision device, providing time for optimization of medical management and volume status to determine the end outcome while allowing her to remain ambulatory. After presentation to the multidisciplinary selection team, she was listed for transplantation as UNOS status 2, not a candidate for durable LVAD placement due to right heart failure.

**Table 1:** Vitals and hemodynamics before and after impella 5.5 with smartAssist.

	Baseline	Pre-explant	1-month outpatient follow-up
Vitals			
Height (cm)	164	164	164
Weight (kg)	98.9	101	92.6
BMI (kg/m^2^)	36.8	37.6	34.4
Hemodynamics			
LVEF (%)	30	53	55
Fick cardiac output (L/min)	4.2	7.5	–
Fick cardiac index (L/min/m^2^)	1.96	3.6	–
Right atrial pressure (mmHg)	17	3	–
Pulmonary artery pressure (mmHg)	42/31	32/13	–
Pulmonary capillary wedge pressure (mmHg)	31	8	–
Blood pressure (mmHg)	76/54	145/83	90/59
MAP (mmHg)	61	104	69
Laboratory values			
Hemoglobin (g/dl)	12	8.7	12.1
Platelets (×10^9^/L)	119	217	358
Lactate dehydrogenase (U/L)	221	803	–
Serum lactate (mmol/L)	0.6	0.7	–
Mixed venous saturation (%)	75.9	76.2	–
Creatinine (g/dl)	1.82	1.37	1.57
eGFR (ml/min/m^2^)	32	45	38
NT-pro BNP (pg/ml)	2,253	–	5,777

**Figure 1:**
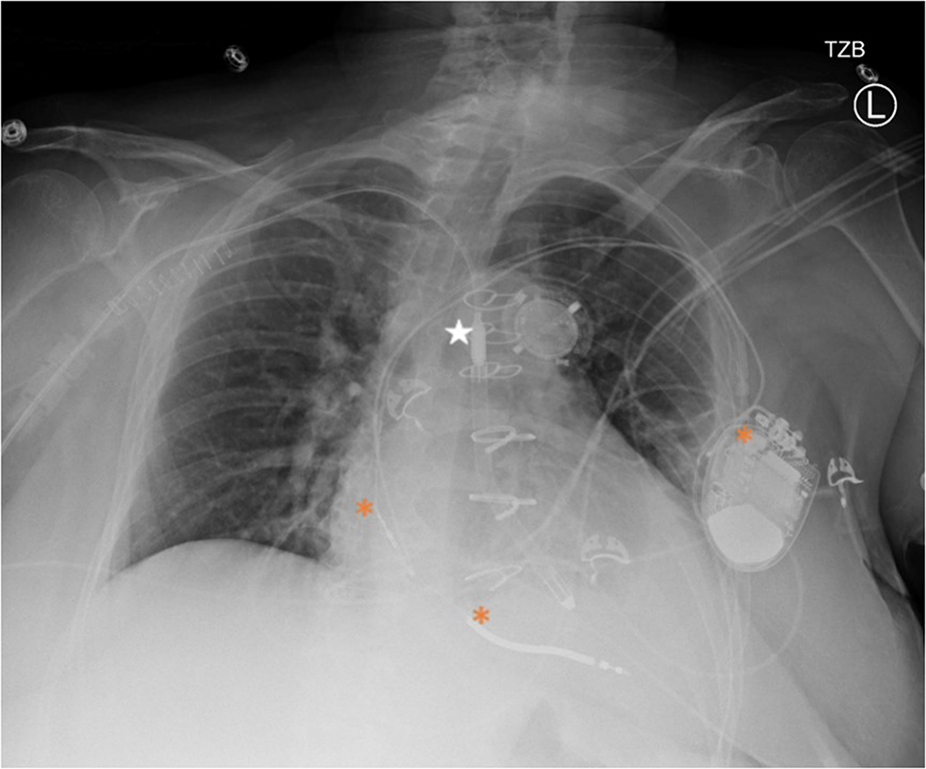
Portable chest x-ray after impella 5.5 placement. Star = Impella 5.5, * = ICD generator and leads in the right atrium and ventricle.

Impella was set to P4 with an estimated flow of 2.4 L/min and remained relatively consistent throughout her hospitalization (Figure 1). Bivalirudin 10.4 mg/hr was initiated anticoagulation.

Re-initiation of medical therapy with entresto 24/26 mg bid, dapagliflozin 10 mg daily, and metoprolol succinate 25 mg daily was slowly performed given progressive rise in systemic blood pressures starting on day 9 of Impella support (BP 148/79 mmHg).

The decision to wean the Impella support was felt to be reasonable for multiple reasons, outlined below, and was further supported by weekly transthoracic echocardiograms (standard protocol at our institution after Impella placement), which demonstrated continuous LVEF improvement, eventually sustained at 50% or greater. Given the concern for unsustained recovery, background Dobutamine 5 mcg/kg/min was added one week before explantation (previously discontinued due to worsening hypertension). Daily echocardiogram-guided wean was performed from P4 to P2.

An acute rise in lactate dehydrogenase (LDH) was seen on impella day 22, suggesting ongoing hemolysis despite systemic anticoagulation, a marker indicating an elevated risk of pump thrombosis. Prior to explantation, invasive hemodynamics showed MAP of 104 mmHg, RA 3 mmHg, PA 32/13 (19) mmHg, PCWP of 8 mmHg, Fick CO of 7.5 L/min and Fick CI of 3.6 L/min/m^2^ (Impella flow of 2.4 L/min). Given her LV recovery and improved end-organ perfusion, the patient underwent device explantation (rather than replacement) on day 23—both due to the risk of Impella failure from progressive LDH rise, and her LV recovery. Bivalirudin was discontinued and subcutaneous heparin was administered. Dobutamine was weaned by 1 mcg/kg/min daily for five days after Impella explant, guided by PICC-based CVP, central mixed saturation, Fick CO/CI, and clinical status. The patient had no signs of worsening renal function and Dobutamine was discontinued successfully. We observed a downward trend in LDH level prior to explant. After Impella explant, LDH was no longer checked due to the inciting factor of hemolysis being removed. The patient was discharged and remained on close follow-up for 1 month before returning home.

Six months after discharge, the patient was removed from UNOS listing due to sustained recovery. One year later, the patient continues to show normalized myocardial function while maintaining previously GDMT as outlined by the American College of Cardiology.

## 3 Discussion

Our case highlights the successful recovery of native left ventricular function in a patient with long-standing ischemic cardiomyopathy (>5 years). In the field of advanced heart failure this is an outlier for multiple reasons. Key factors identified in this specific case, and may be evident in other patients, are increased baseline biventricular filling pressures with low cardiac output and inability to offload the patient with inotrope and GDMT.

GDMT as outlined by the American College of Cardiology for the management of heart advanced failure and coronary artery disease as well as advanced therapies such as coronary artery bypass grafting in 3 vessels (6, 7) was unsuccessful in mitigating elevation in intracardiac filling pressures, renal optimization, and myocardial recovery (either as an improvement in LV function or decrease in LV cavity size). Despite timely intervention, many patients with ischemic cardiomyopathy exhibit some range of chronic HF symptoms, with many progressing to advanced heart failure and cardiogenic shock. Traditionally, patients with HF-CS require immediate hospitalization to optimize fluid status, biventricular function and preserve end organ function with medical management and consideration for advanced therapies. Hernandez-Montfort et al. reported that patients in SCAI stage D had a 29.4% mortality rate and 26.6% survival without a durable VAD or transplant (8). Despite the prevailing belief that heart failure stemming from ischemic causes is predominantly irreversible, our remarkable results show the significance of early mechanical circulatory support indicating that there is great potential for sustained LV recovery even in cases of prolonged ischemic injury leading to chronic heart failure culminating in cardiogenic shock (HF-CS). Consideration should be taken in the current state of heart failure management to consider the optimal way to improve both quality and quantity of patient lives—given the shortage of available donor organs, limited survival post-durable LVAD and increased risk of post-transplant related complications (e.g., cancer, infection, rejection) (9–11).

### 3.1 Impella management in the ICU

The daily management of the Impella 5.5 device in patients with HF-CS varies throughout hospital settings. Our practice is to initiate the patient on Impella support at power (P) levels between 6 and 8, achieving 3–5 L/min of support when leaving the operating room and awaiting extubation. Post-extubation, we maintain patients on the lowest tolerable P level for a few reasons: (1) to decrease suction events and LV irritability, (2) To allow low-levels of background vasoactive support for right heart function (e.g., milrinone 0.25 mcg/kg/min or dobutamine 2.5–5 mcg/kg/min), and (3) to allow more support if needed without needing to escalate to peripheral or central VA-ECMO.

Furthermore, our patients are systemically anticoagulated using bivalirudin to avoid pre-transplant or LVAD exposure to heparin and decrease their chances of developing heparin induced thrombocytopenia or thrombosis. For patients awaiting transplant, transfusion thresholds are set at 8 gm/dl of hemoglobin or lower, to prevent sensitizing events.

Weekly assessment with trans-thoracic echocardiogram is performed to look at distance from aortic annulus, mitral apparatus involvement, and left and right ventricular function, given the increased mobility of our patients. All patients at our institution initiate ambulation within 24 h of device placement, often ambulating between 3 and 10 miles per day while on support (12). Daily LDH is assessed to monitor for pump thrombosis, as plasma free hemoglobin requires 7 days to return to our facility. Right heart catheterization is maintained for 72 h after Impella placement and re-assessment of cardiopulmonary filling pressures is performed if patients are refractory to medical and mechanical support or have a clinical or functional decline.

### 3.2 Role of early mechanical circulatory support

MCS devices improve LV function via augmentation of forward blood flow which ultimately results in reducing LV filling pressures and enhancing coronary and end-organ perfusion in patients with CS refractory to standard medical management (3).

The progressively declining LVEF and steadily rising NT-pro-BNP with high pulmonary pressures in our patient warranted urgent intervention in this case to prevent a fatal outcome. The utilization of axillary MCS with Impella 5.5 allowed us time to determine the best course of action for this patient. Durable LVAD does add some patient years prior to transplant as reported. However, these are often complicated with LVAD related bleeding, stroke, or infection. Impella as a bridge to decision for advanced therapies or native left ventricular recovery is safe for a prolonged period of time and allows an immediate cessation of the devastation that comes along with cardiogenic shock. We highlight this case to demonstrate the potential for sustained left ventricular recovery despite chronic ischemic heart failure. The Impella 5.5 provided a platform for sustained left ventricular offloading and decongestion, recoupling of her left ventricular—aortic axis, while maintaining her ability to rehabilitate and tolerate safe re-initiation of GDMT without end-organ hypoperfusion.

Currently, a scientific statement from the American Heart Association (AHA) on the escalation and de-escalation of temporary MCS use in patients with cardiogenic shock exists for guiding practice patterns. In HF-CS refractory to inotropes and pressors, persistently low CI < 2.2 L/min/m^2^, evidence of hypoperfusion (increasing lactate, worsening renal function), and MAP < 65 mmHg, one should consider escalation to temporary MCS (10). Our patient had persistently low CI (Table 1) and was intolerant to escalating doses of vasoactive agents due to refractory atrial and ventricular arrhythmias. Our protocol to determine appropriate device selection has previously been published (13).

Weaning practices are variable between institutions and generally are guided by provider comfort and patient response. Daily multi-disciplinary rounds between critical care, transplant cardiology, cardiothoracic surgery and transplant nephrology were undertaken and the decision to wean and explant the Impella was based on the de-escalation guidelines set by the AHA and our patients clinical condition: resolution of end-organ perfusion, MAP ≥ 65 mmHg, RAP ≤ 10–15 mmHg, PCWP ≤ 18 mmHg, CI > 2.2 L/min/m^2^, lactate <2.0 mmol/L, LVEF > 20%–25% and hemodynamic stability on dobutamine support (10). During the weaning process, however, our contingency was set that if any of these hemodynamic values remained abnormal, then the patient would have been deemed unsuitable for wean and would continue to remain listed as UNOS Status 2 for heart transplantation (designated by the UNOS criteria for any patient with MCS and shock criteria prior to the implant of their device).

### 3.3 LV recovery in chronic heart failure

While left ventricular recovery outcome data remains limited, numerous single-centre studies have shown favourable outcomes with the use of Impella devices in patients with CS. Our group has reported comparable results, demonstrating a 95% 1-year survival rate among post-transplant patients who received an average of 27 days of Impella support prior to heart transplantation (13). While FDA-approved for ≤14 days, Carlos et al. corroborated our published data, reporting that prolonged impella use, even in high-risk patients with cardiogenic shock does not increase the risk of complications or mortality (14).

The improved functionality observed in cardiac myocytes with MCS support in failing hearts stems from improved myocyte contractility and enhanced myocyte relaxation compared to patients without MCS bridging. Molecular mechanisms, such as increased beta receptor density and enhanced calcium handling, are also cited as contributing factors to left ventricular recovery (15).

Underlying mechanisms such as these are further supported by the clinical response we saw during our patient's course—reducing myocardial stress, improving LV-Aortic coupling, and maintaining renal perfusion—all likely affecting the renin-angiotensin-aldosterone axis and balancing sympathetic and parasympathetic systems, aiding to the synergistic effect of initiating GDMT when early signs of left ventricular recovery were noted.

This case signifies that early recognition of patient profiles that may benefit from temporary MCS offloading is crucial to a successful and sustained recovery of left ventricular function. Further insight from our case has the potential to bear significant future implications for the emergence and use of MCS devices in advanced heart failure. This is more prudent when considering a 66% forecasted rise in the prevalence of heart failure by 2030 (16).

## 4 Conclusion

This case allows us to question the conventional belief that a failing heart, particularly after a long-standing cardiomyopathy, cannot recover its native function. We offer a novel perspective, supported by multiple measurements such as ejection fraction, renal function, and cardiac index—all indicating that sustained native heart recovery in a patient with chronic heart failure is both feasible and safe when the use of Impella 5.5 is applied early.

## Data Availability

The raw data supporting the conclusions of this article will be made available by the authors, without undue reservation.

## Supplementary material

Supplementary Figure S1 De-escalation (impella weaning) guidelines outlined by the American Heart Association [9]. ECMO, indicates extracorporeal membrane oxygenation; LV, left ventricular; LVEF, left ventricular ejection fraction; LVOT, left ventricular outflow tract; MAP, mean arterial pressure; MCS, mechanical circulatory support; PAPi, pulmonary artery pulsatility index; PCWP, pulmonary capillary wedge pressure; RA, right atrial; RV, right ventricular; RVEF, right ventricular ejection fraction; tMCS, temporary mechanical circulatory support; and VTI, velocity-time integral. *No single metric should be used in isolation to determine weaning suitability.
