# Supplementary figures and images for: Case Report: Unmasking sustainable left ventricular recovery in chronic heart failure with axillary temporary mechanical circulatory support
Source: Front Cardiovasc Med. 2024 Aug 27;11:1407552. doi: 10.3389/fcvm.2024.1407552 (PMC11385610; doi:10.3389/fcvm.2024.1407552)

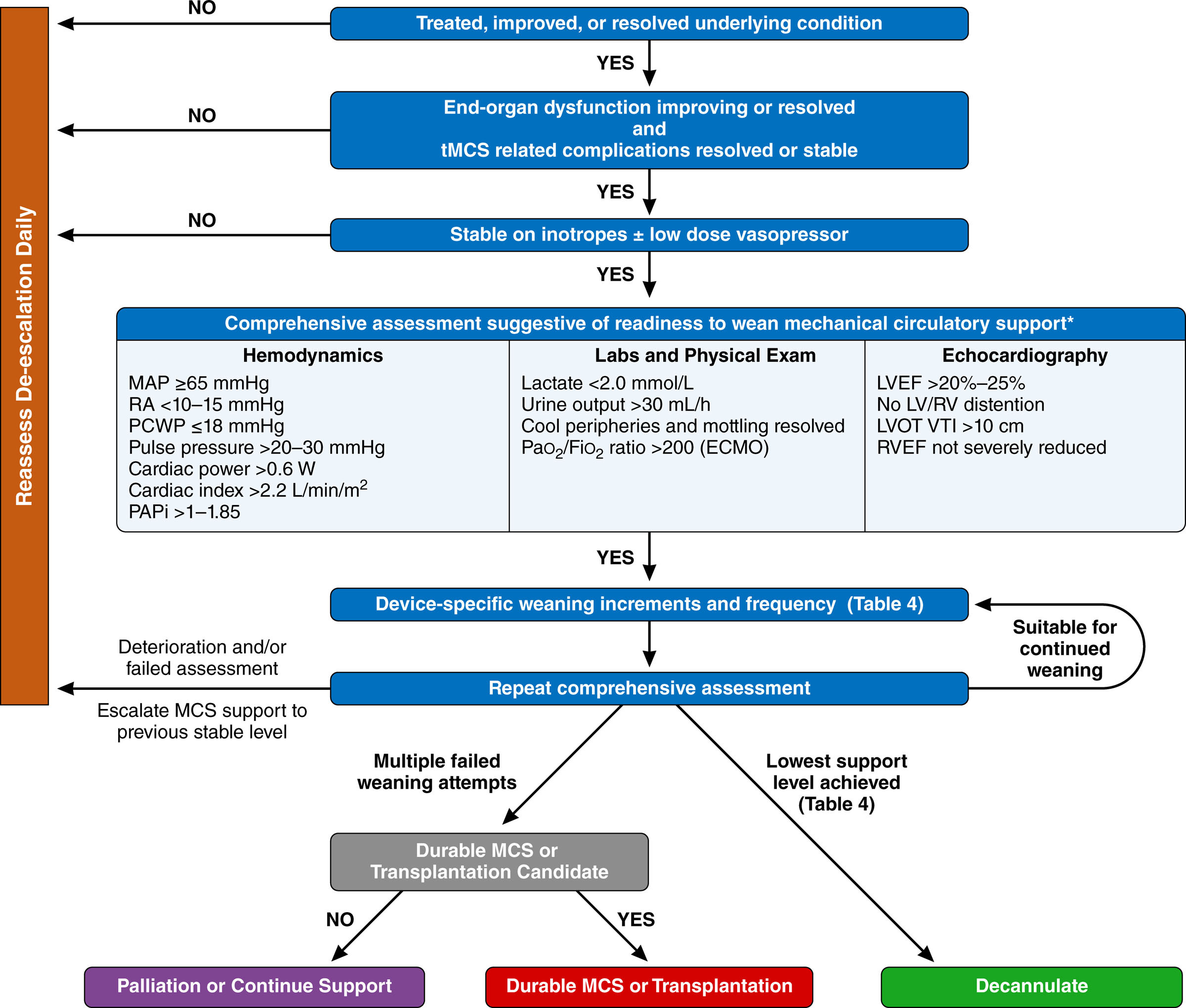

Supplement: Supplementary Figure S1 — De-escalation (impella weaning) guidelines outlined by the American Heart Association [9]. ECMO, indicates extracorporeal membrane oxygenation; LV, left ventricular; LVEF, left ventricular ejection fraction; LVOT, left ventricular outflow tract; MAP, mean arterial pressure; MCS, mechanical circulatory support; PAPi, pulmonary artery pulsatility index; PCWP, pulmonary capillary wedge pressure; RA, right atrial; RV, right ventricular; RVEF, right ventricular ejection fraction; tMCS, temporary mechanical circulatory support; and VTI, velocity-time integral. *No single metric should be used in isolation to determine weaning suitability. [file Image1.jpeg]
